# Contrast-Enhanced Ultrasound-Guided Liver Tumor Biopsy for Primary Hepatic Angiosarcoma: A Case Series

**DOI:** 10.7759/cureus.91787

**Published:** 2025-09-07

**Authors:** Yamasaki Shintaro, Fujii Yasutoshi, Tsuge Masataka, Arihiro Koji, Oka Shiro

**Affiliations:** 1 Department of Gastroenterology and Metabolism, Hiroshima University Hospital, Hiroshima, JPN; 2 Department of Gastroenterology and Metabolism, Hiroshima University, Hiroshima, JPN; 3 Liver Center, Hiroshima University Hospital, Hiroshima, JPN; 4 Department of Anatomical Pathology, Hiroshima University, Hiroshima, JPN; 5 Department of Endoscopy, Hiroshima University Hospital, Hiroshima, JPN

**Keywords:** contrast-enhanced ultrasound, histopathological diagnosis, liver tumor biopsy, primary hepatic angiosarcoma, rare malignant liver tumor

## Abstract

Primary hepatic angiosarcoma (PHA) is a rare and aggressive malignant liver tumor that is often difficult to diagnose due to its nonspecific clinical presentation and imaging findings. This case series aimed to illustrate the utility of contrast-enhanced ultrasound (CEUS)-guided percutaneous liver biopsy for the definitive diagnosis of unresectable PHA. We report three patients who presented with nonspecific symptoms and hepatic masses. Imaging findings were inconclusive for a definitive diagnosis, and histopathological evaluation was required. All patients underwent CEUS-guided percutaneous liver tumor biopsy. CEUS enabled real-time visualization of viable tumor regions, facilitating accurate needle placement and minimizing sampling from necrotic or hemorrhagic areas.

In Case one, multiple punctures were needed to obtain viable tissue due to extensive necrosis. In Cases two and three, CEUS was particularly useful for demarcating poorly defined lesions, resulting in successful tissue sampling with fewer punctures. Biopsies were performed during the post-vascular phase, enhancing diagnostic yield. Histopathological analysis revealed CD31-positive spindle-shaped cells and a high Ki-67 index, confirming the diagnosis of PHA in all cases. All patients died within six months of diagnosis despite chemotherapy, underscoring the poor prognosis and clinical urgency.

CEUS-guided percutaneous liver biopsy is a minimally invasive and reliable technique for the diagnosis of PHA, especially in patients who are not candidates for surgery. This approach enables targeted tissue acquisition, potentially improves diagnostic accuracy, and may contribute to the earlier management of this rare malignancy.

## Introduction

Primary hepatic angiosarcoma (PHA) is an extremely rare and aggressive malignant liver tumor, accounting for less than 1% of all primary hepatic malignancies and 1-2% of all soft tissue sarcomas [1]. Although it more commonly affects older men and is associated with exposure to vinyl chloride, thorium dioxide, arsenic, and radiation [1-3], most cases are idiopathic [4]. Clinical symptoms are variable and mimic chronic liver disease. They include abdominal pain, anorexia, fatigue, weight loss, fever, and low back pain, and may even be asymptomatic [5]. In advanced stages, acute life-threatening hemorrhage from hepatic tumor rupture or liver failure can occur [5,6].

Because PHA lacks specific clinical manifestations, laboratory abnormalities, and imaging features, it remains challenging to diagnose in clinical settings and is frequently misdiagnosed as hepatic hemangioma or primary hepatocellular carcinoma [7]. Imaging alone is insufficient for definitive diagnosis, and histopathological evaluation is essential [8,9]. In cases where surgery is not indicated, percutaneous liver biopsy is commonly performed. However, due to hypervascularity and necrosis within the fragile tumor, acquiring adequate viable tissue for biopsy can be technically difficult and increases the risk of false-negative results and hemorrhagic complications [9,10].

Contrast-enhanced ultrasound (CEUS) facilitates the detection of poorly demarcated lesions and provides dynamic perfusion information, enabling real-time visualization of viable tumor regions while avoiding necrotic areas during biopsy. This approach enhances diagnostic accuracy and may also help reduce biopsy-related complications [9]. Nevertheless, the bleeding risk remains in highly vascular tumors, and pre-procedural assessment of coagulation status is essential. Although several reports have described CEUS-guided biopsy for PHA, few have unambiguously specified the contrast-enhancement phase during which the biopsy was performed. We herein report three cases of liver tumors that were successfully diagnosed with PHA by CEUS-guided percutaneous transhepatic liver tumor biopsy.

## Case presentation

Three patients who underwent CEUS-guided biopsy were diagnosed with PHA at our hospital between April 2020 and March 2025 (Table 1). PHA was suspected based on laboratory and imaging findings, but imaging findings alone were inconclusive, and histopathological examination was required to confirm the diagnosis. All examinations were performed after obtaining written informed consent from patients. This study was approved by the Ethics Committee of Hiroshima University and conducted by the principles outlined in the Declaration of Helsinki.

**Table 1:** Patient characteristics AE: Adverse event, BSC: Best supportive care, C: Cycle, D: Day, PS: Performance status, PTX: Paclitaxel

Case	Age	Gender	Initial (presenting) symptoms	Tumor characteristics	Diagnostic Devices	Treatment	Prognosis
								1	72	Female	Appetite loss and generalized fatigue	Single on the right lobe	BioPince^®^ Ultra18G	Weekly PTX (80 mg/m²)	Died five months after diagnosis
→ C3D1: BSC															
total 5 times	(Due to neutropenia and PS decline)														
2	66	Female	Fever and abdominal distension	Diffuse on the right lobe	BioPince^®^ Ultra18G	Weekly PTX (80 mg/m²)	Died two months after diagnosis
						→ D5: BSC	
					total 4 times	(Due to ascites, neutropenia, and PS decline)	
3	77	Male	Asymptomatic	Multiple on both lobes	BioPince^®^ Ultra18G	Weekly PTX (80 mg/m²)	Died three months after diagnosis
						→ 60 mg/m² from C2 (Due to neutropenia)	
					total 2 times	→ Post-C2: BSC (Due to liver failure)	

Case one

A 72-year-old woman with a history of appendectomy was referred to our hospital because of appetite loss and generalized fatigue. An 8-cm mass was detected in the right lobe of the liver. She didn’t have a history of exposure to vinyl chloride or other toxins. Laboratory examinations revealed elevated liver enzymes, lactate dehydrogenase, and C-reactive protein (CRP), with decreased albumin (Table 2). Tumor markers showed elevated carbohydrate antigen 19-9 (CA19-9) (671.4 U/mL), while carcinoembryonic antigen (CEA), alpha-fetoprotein (AFP), and des-gamma-carboxy prothrombin (DCP) were within normal limits. Viral markers for hepatitis B virus (HBV) and hepatitis C virus (HCV) were negative.

**Table 2:** Laboratory examinations AFP: Alpha-fetoprotein, Alb: Albumin, ALP: Alkaline phosphatase, ALT: Alanine aminotransferase, APTT: Activated partial thromboplastin time, AST: Aspartate aminotransferase, BUN: Blood urea nitrogen, CA19-9: Carbohydrate antigen 19-9, CEA: Carcinoembryonic antigen, Cre: Creatinine, CRP: C-reactive protein, DCP: Des-gamma-carboxy prothrombin, γ-GTP: Gamma-glutamyl transpeptidase, Hb: Hemoglobin, HBs antigen: Hepatitis B surface antigen, HBs antibody: Hepatitis B surface antibody, HCV antibody: Hepatitis C virus antibody, IgG HBc antibody: Immunoglobulin G Hepatitis B core antibody, LDH: Lactate dehydrogenase, PLT: Platelet count, PT: Prothrombin time, PT-INR: Prothrombin time-international normalized ratio, RBC: Red blood cell count,sIL-2R: Soluble interleukin-2 receptor, T-bil: Total bilirubin, TP: Total protein, WBC: White blood cell count

	Reference range	Case 1	Case 2	Case 3
WBC (/μL)	3300-8600	6370	7530	5740
RBC (10^4^/μL)	386-492	262	354	461
Hb (g/dL)	11.6-14.8	7.5	11.7	14.7
PLT (10^3^/μL)	158-348	281	105	215
T-Bil (mg/dL)	0.4-1.5	1.5	1.5	1.6
AST (U/L)	13-30	81	114	26
ALT (U/L)	7-23	42	72	25
LDH (U/L)	124-222	833	998	194
ALP (U/L)	106-322	480	138	165
ɤ-GTP (U/L)	9-32	297	641	77
TP (g/dL)	6.6-8.1	7.2	6.8	7.8
Alb (g/dL)	4.1-5.1	2.8	3.1	4.0
BUN (mg/dL)	8-20	10.9	32.3	15.8
Cre (mg/dL)	0.46-0.79	0.63	1.36	0.81
CRP (mg/dL)	0-0.14	13.17	3.83	0.38
PT-INR	0.80-1.20	1.27	1.23	1.09
APTT (sec)	26.9-38.1	28.8	33.9	30.4
D-dimer (μg/mL)	0.4-1.5	31.7	14.1	1.7
AFP (ng/mL)	0-10	1.8	10.5	1.4
DCP (mAU/mL)	0-40	24	44	15
CEA (ng/mL)	0-5	1.5	2.4	3.1
CA19-9 (U/mL)	0-37	671.4	0.5	0.5
sIL-2R (U/mL)	121-613	782	1479	261
HBs antigen	(-)	(-)	(-)	(-)
HBs antibody	(-)	(-)	(-)	(-)
IgG HBc antibody	(-)	(-)	(-)	(-)
HCV antibody	(-)	(-)	(-)	(-)

**Figure 1:**
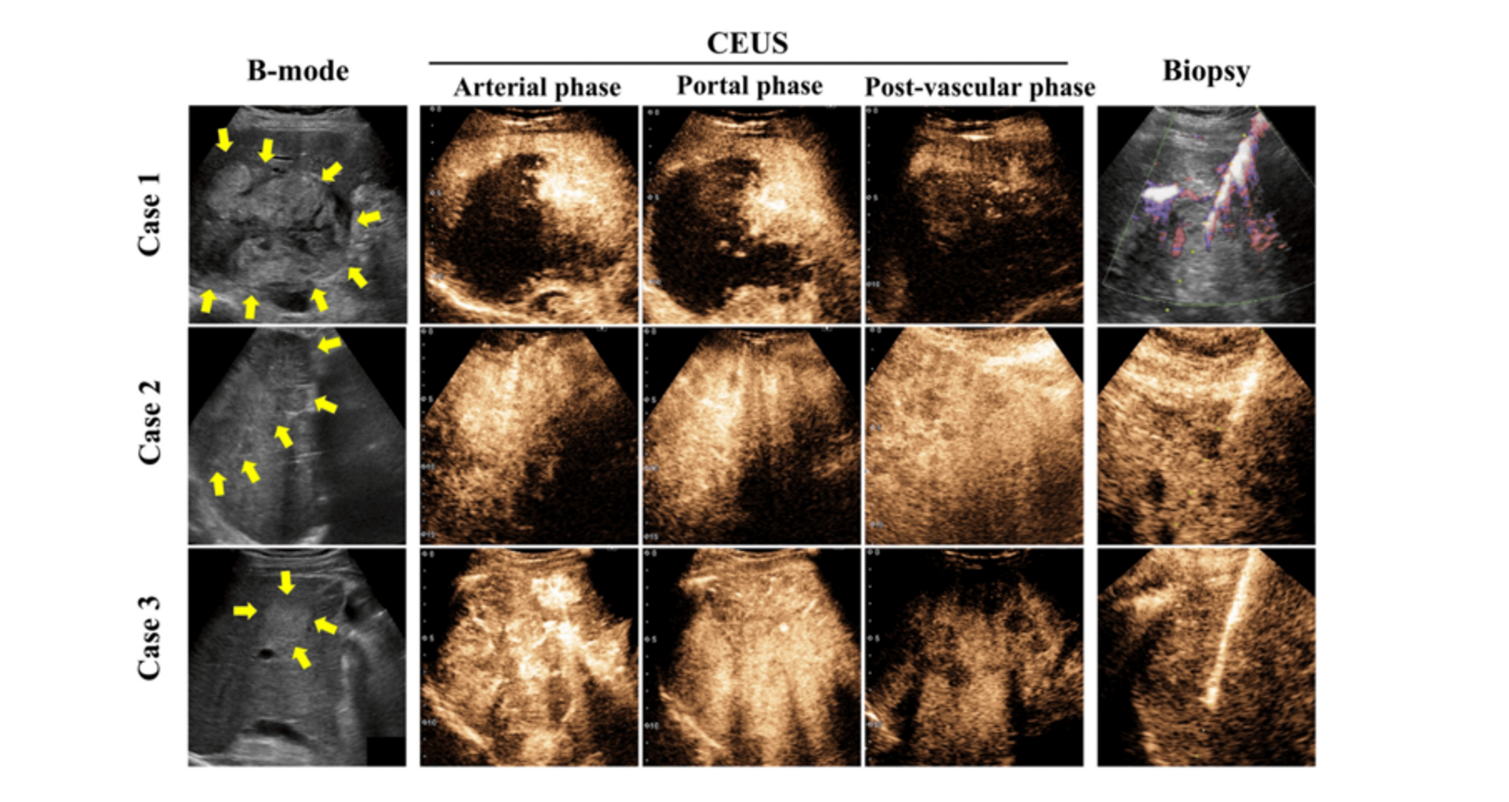
B-mode and contrast-enhanced ultrasound (CEUS) images B-mode and contrast-enhanced ultrasound (CEUS) images in each of the three patients. Images are shown for the arterial phase (15 seconds), portal venous phase (90 seconds), post-vascular phase (15 minutes), and during CEUS-guided percutaneous liver tumor biopsy.

Imaging studies showed a mosaic-patterned mass on B-mode ultrasonography. CEUS demonstrated peripheral early enhancement with a defect in the post-vascular phase (Figure 1).

**Figure 2:**
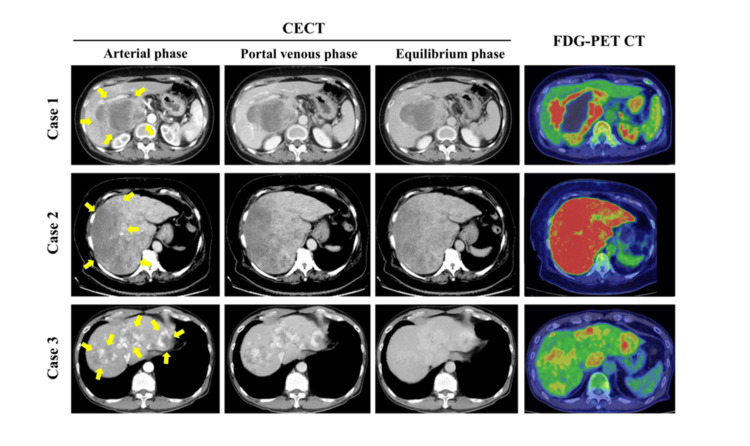
CT findings Dynamic contrast-enhanced computed tomography CT (CTCE) at the arterial phase, portal venous phase, and equilibrium phase are shown, as well as 18F-fluorodeoxyglucose positron emission tomography CT (FDG-PET CT) findings.

**Figure 3:**
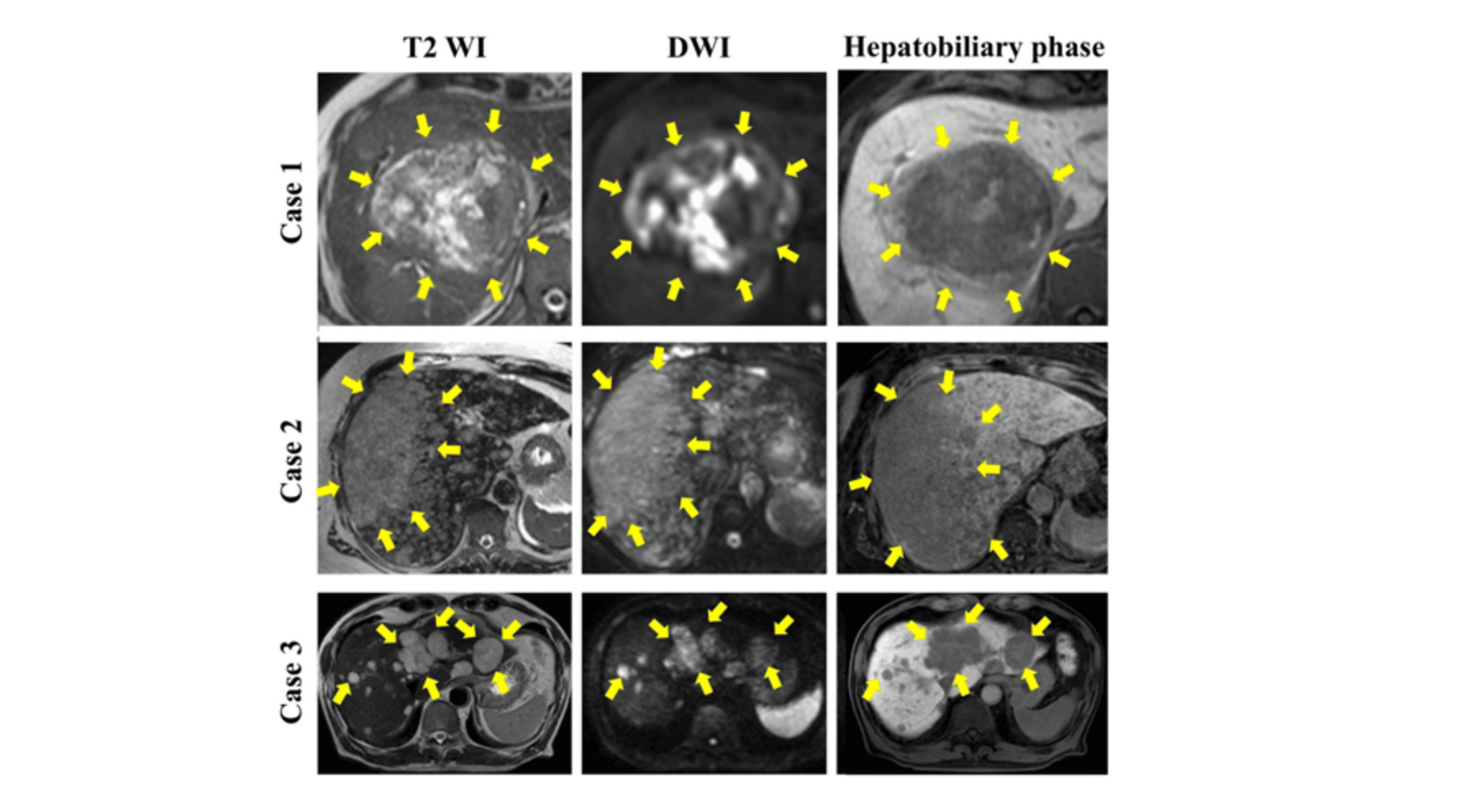
MRI findings Gadolinium ethoxybenzyl-diethylenetriaminepentaacetic acid (Gd-EOB-DTPA) enhanced MRI findings. T2-weighted images with fat saturation (T2WI-FS), diffusion-weighted image (DWI), and hepatobiliary phase are shown.

Dynamic contrast-enhanced computed tomography (CECT) revealed peripheral enhancement, and magnetic resonance imaging (MRI) with gadolinium-ethoxybenzyl-diethylenetriamine pentaacetic acid (Gd-EOB-DTPA) showed a hyperintense lesion on fat-suppressed T2-weighted image (T2WI-FS) and a hypointense lesion in the hepatobiliary phase (Figures 2, 3). Fluorodeoxyglucose-positron emission tomography (FDG-PET) revealed strong uptake (maximum standardized uptake value (SUVmax) 17.2) with central areas of necrosis (Figure 2).

**Figure 4:**
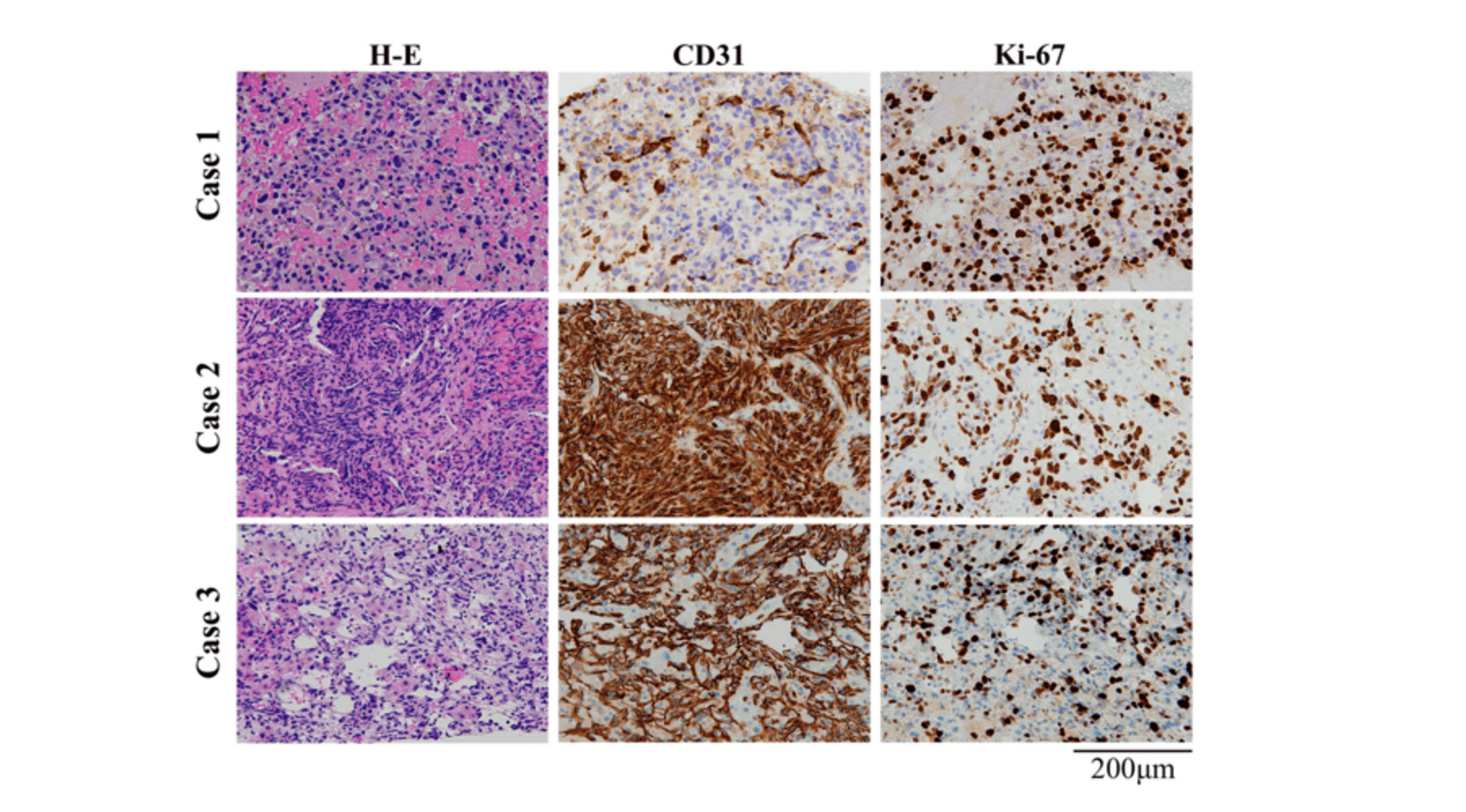
Histopathological findings Hematoxylin and eosin staining (H-E) and immunohistochemical staining for CD31 and Ki-67 were performed. All images were obtained at ×200 magnification.

Imaging findings suggested an advanced malignant liver tumor, with differential diagnoses including poorly differentiated hepatocellular carcinoma, intrahepatic cholangiocarcinoma, primary hepatic angiosarcoma, and malignant lymphoma. However, histopathological examination was essential for establishing the definitive diagnosis. Diagnostic surgical resection was contraindicated due to inferior vena cava invasion. Tumor biopsies were performed with CEUS, which allows for the precise localization of viable tumors to ensure sufficient tissue volume for diagnosis. CEUS-guided biopsy was performed using an 18-gauge BioPince Ultra needle (Argon Medical Devices, Plano, TX, US) during the post-vascular phase. Despite targeting viable regions, necrotic tissue was obtained in several attempts, and a total of five punctures were required. Histopathological examination revealed spindle-shaped tumor cells with CD31 positivity, while CK AE1/AE3, Cam5.2, EMA, S100, αSMA, hepatocyte, glypican-3, arginase, CD34, D2-40, and CD68 were all negative, and the Ki-67 index wa approximately 70% (Figure 4).

The diagnosis of hepatic angiosarcoma was made based on the pathological findings. She received systemic chemotherapy with paclitaxel but died five months after diagnosis.

Case two

A 66-year-old woman with a history of breast and bladder cancer treated by curative surgery and chemoradiotherapy, and comorbidities, including hypertension, dyslipidemia, type 2 diabetes mellitus, and obesity (body mass index 34 kg/m²), presented with a fever and abdominal distension. Diffuse hepatic tumors were detected at her first visit. She didn’t have a history of exposure to vinyl chloride or other toxins. Laboratory findings showed elevated liver enzymes, lactate dehydrogenase, and CRP, with decreased albumin (Table 2). Tumor markers (CEA, CA19-9, AFP, and DCP) were all within normal ranges. Viral markers for HBV and HCV were negative. Imaging demonstrated diffuse, poorly demarcated lesions on B-mode ultrasound. Although early enhancement was unclear on dynamic CECT, CEUS revealed enhancement in the arterial phase with persistent enhancement in the portal phase (Figures 1, 2). MRI with gadolinium ethoxybenzyl-diethylenetriaminepentaacetic acid (Gd-EOB-DTPA) revealed hyperintensity on T2WI-FS and hypointensity in the hepatobiliary phase (Figure 3). FDG-PET showed high uptake with SUVmax 14.3 (Figure 2). Imaging findings suggested an advanced malignant liver tumor, with differential diagnoses. However, histopathological examination was essential for establishing the definitive diagnosis. Diagnostic surgical resection was contraindicated due to diffuse invasion throughout the liver. Tumor biopsies were performed with CEUS, which allows for the precise localization of viable tumors to ensure sufficient tissue volume for diagnosis. Biopsy was performed using CEUS guidance during the post-vascular phase, targeting areas of perfusion defect. Three punctures were required to obtain adequate viable tissue. Histopathology revealed spindle-shaped tumor cells that were positive for CD31 and CD34, and negative for D2-40 and Factor VIII, with a Ki-67 index of approximately 80% (Figure 4). The diagnosis of hepatic angiosarcoma was made based on the pathological findings. She received systemic chemotherapy with paclitaxel but died two months after diagnosis.

Case three

A 77-year-old man with a history of osteoporosis was asymptomatic, but a pathological fracture due to osteoporosis prompted further evaluation. During imaging studies for the fracture, multiple hepatic masses in both lobes and multiple bone metastases were incidentally detected. He didn’t have a history of exposure to vinyl chloride or other toxins. Laboratory data showed mildly elevated liver enzymes, while tumor markers (CEA, CA19-9, AFP, and DCP) were within normal limits (Table 2). Viral markers for HBV and HCV were negative. Ultrasound revealed multiple slightly hyperechoic nodules with poorly defined margins. CEUS showed early enhancement and a post-vascular phase defect (Figure 1). CECT demonstrated heterogeneous enhancement, and MRI revealed hyperintensity on T2WI-FS and hypointensity in the hepatobiliary phase (Figures 2, 3). FDG-PET revealed uptake with SUVmax 8.8 (Figure 2). Imaging findings suggested an advanced malignant liver tumor, with differential diagnoses. However, histopathological examination was essential for establishing the definitive diagnosis. Diagnostic surgical resection was contraindicated due to bone metastases. Tumor biopsies were performed with CEUS, which allows for the precise localization of viable tumors to ensure sufficient tissue volume for diagnosis. CEUS-guided biopsy was performed during the post-vascular phase. Two punctures with an 18-gauge needle provided sufficient viable tissue for diagnosis. Histopathology demonstrated spindle-shaped tumor cells positive for CD31 and CD34, negative for D2-40, with a Ki-67 index of approximately 70% (Figure 4). The diagnosis of hepatic angiosarcoma was made based on the pathological findings. He received systemic chemotherapy with paclitaxel, but died three months after diagnosis.

Table 3 summarizes the imaging findings of the three cases. In all cases, imaging findings suggested an advanced malignant liver tumor, with differential diagnoses including poorly differentiated hepatocellular carcinoma, intrahepatic cholangiocarcinoma, primary hepatic angiosarcoma, and malignant lymphoma. However, histopathological examination was essential for establishing the definitive diagnosis. In all cases, surgical treatment was not feasible, and percutaneous liver tumor biopsy was chosen.

**Table 3:** Imaging findings CECT: contrast-enhanced computed tomography, CEUS: contrast-enhanced ultrasound, DWI: diffusion-weighted imaging, FDG-PET: fluorodeoxyglucose-positron emission tomography, Gd-EOB-DTPA: gadolinium-ethoxybenzyl-diethylenetriamine pentaacetic acid, MRI: magnetic resonance imaging, SUV: standardized uptake value; T1WI: T1-weighted Image, T2WI-FS: fat-suppressed T2-weighted imaging

Case	B-mode	CEUS			CECT			Gd-EOB-DTPA enhanced MRI				FDG-PET
													Arterial	Portal	Post-vascular	Arterial	Portal	Equilibrium	T1 WI	T2 WI-FS	DWI	Hepatobiliary
		phase																			
		1	Mosaic-	Peripheral enhancement	Persistent	Defect	Peripheral enhancement	Persistent	Persistent	Low	High		High	Low	SUV max 17.2
patterned	enhancement		enhancement		enhancement			Central necrosis						
2	Mosaic-	Peripheral enhancement	Persistent	Defect	Unclear enhancement	Low	Low	Low	High	High	Low	SUV max 14.3
	patterned		enhancement									
	Poorly demarcated											
3	Slightly hyperechoic	Peripheral	Iso-	Defect	Mottled enhancement	Persistent	Persistent	Low	High	High	Low	SUV max 8.8
	Poorly demarcated	enhancement	echogenicity			enhancement	enhancement					

A CEUS-guided percutaneous liver tumor biopsy was performed in all cases using a diagnostic ultrasound system (Aplio i900, Canon Medical Systems, Japan) with a convex probe (PVI-475BX; frequency range: 1.8-6.2 MHz, center frequency: ~3.5 MHz). A perflubutane microbubble contrast agent (Sonazoid, GE Healthcare Japan, Hino, Japan) was administered intravenously at a dose of 0.6 mL/body, followed by a 10 mL saline flush. The mechanical index was set to 0.17-0.22, and the dynamic range was 3.3-3.5. Tumor puncture was performed during the post-vascular phase, targeting viable tumor tissue identified on the arterial phase. Because it is technically difficult to puncture lesions during the arterial phase due to the very short time window, biopsies were performed during the post-vascular phase. At this phase, regions that appeared hyper-enhancing in the arterial phase and then became hypo-enhancing could be clearly identified as viable tumor areas, allowing us to target active tissue more precisely and avoid necrotic regions. An 18-gauge semi-automatic biopsy needle (BioPince Ultra) was used for the tumor biopsy.

Five punctures were performed in Case one, three in Case two, and two in Case three. In Case one, after identifying a viable area using CEUS, punctures were performed to avoid the vessels surrounding the tumor on color Doppler images. Nevertheless, in three of the attempts, only coagulated/necrotic tissue was collected, and a total of five punctures were required. In cases two and three, because of the poorly demarcated boundary between normal liver and tumor, the punctures were performed targeting the areas in which defects were detected during the post-vascular phase, allowing sufficient tissue to be collected in both cases (Figure 1). In all cases, background liver biopsies were also performed, revealing no evidence of significant fibrosis or inflammatory cell infiltration. All patients received systemic chemotherapy with paclitaxel following the histopathological diagnosis. Despite treatment, Cases one, two, and three died five, two, and three months after diagnosis, respectively.

## Discussion

In this case series, we report three patients who were successfully diagnosed with PHA based on CEUS-guided percutaneous liver tumor biopsy. In all cases, surgical resection was not feasible due to extrahepatic metastases, poor performance status, or extensive intrahepatic involvement at the time of diagnosis.

A definitive diagnosis is often difficult due to nonspecific clinical presentation and imaging findings that resemble other hepatic tumors [7], but some reports have presented imaging findings characteristic of PHA. On conventional ultrasonography, PHA lesions are typically irregular in shape, have ill-defined margins, and show heterogeneous internal echogenicity without a surrounding capsule [11], reflecting their infiltrative growth pattern and structural heterogeneity. CEUS has been reported to demonstrate distinctive features of PHA, such as rim-like arterial enhancement and the characteristic “black hole sign” in the post-vascular phase [12]. CECT and MRI findings are variable, often demonstrating arterial phase hypervascular enhancement, reflecting the heterogeneous vascularity of PHA, and FDG-PET often shows increased uptake due to high metabolic activity [13-17]. These findings, although not pathognomonic, were consistent with our cases and raised suspicion for PHA. However, they were insufficient for a definitive diagnosis, highlighting the need for histopathological confirmation. Thus, histopathological confirmation was pursued via CEUS-guided percutaneous biopsy. CEUS enabled real-time identification of viable tumor tissue, allowing targeted sampling during the post-vascular phase while avoiding necrotic or hemorrhagic areas. In Case one, CEUS facilitated needle placement by clearly delineating viable regions and avoiding areas of necrosis and hemorrhage. In Cases two and three, CEUS improved the visibility of lesions that were poorly defined on conventional ultrasound, enabling accurate targeting and reducing the number of punctures required to obtain a diagnostic sample. This approach minimized the number of punctures and improved diagnostic yield. Previous reports of CEUS-guided biopsy in hepatic angiosarcoma have been limited and often did not specify the contrast-enhancement phase used for tissue acquisition. Our study highlights not only the feasibility but also the importance of performing a biopsy during the post-vascular phase, which may improve diagnostic yield and safety. Our findings suggest that CEUS-guided biopsy is an effective diagnostic method, particularly for patients who are not surgical candidates. This technique facilitates real-time targeting of viable tumor regions, potentially reducing complications and enhancing diagnostic accuracy. Given the poor prognosis and lack of standard therapies, prompt and accurate diagnosis is essential. All three patients died within six months of diagnosis, underscoring the critical importance of prompt and accurate diagnosis in such patients. Accumulation of biopsy-confirmed cases with detailed radiologic-pathologic correlation will be important in guiding future diagnostic and therapeutic strategies. In addition, further studies are warranted to explore novel therapeutic approaches and to evaluate the potential role of CEUS-guided biopsy not only for diagnosis but also for monitoring treatment response and detecting disease progression.

This study has several limitations. First, it is a small case series of only three patients, and the findings may not be generalizable to all patients with PHA. Second, biopsy procedures and imaging interpretations were performed at a single institution, which may limit reproducibility. Third, clinical outcomes were poor despite diagnosis, and our report does not address the efficacy of therapeutic options. Larger, multi-center studies are needed to validate the role of CEUS-guided biopsy and to establish standardized diagnostic strategies, which may ultimately improve the management and outcomes of patients with this rare and aggressive tumor.

## Conclusions

CEUS-guided percutaneous liver biopsy is a minimally invasive and reliable diagnostic option for PHA. It enables accurate tissue acquisition even in high-risk patients, potentially facilitating earlier diagnosis and improved clinical management of this rare malignancy.

Given the limited treatment options and poor prognosis of PHA, prompt histopathological confirmation is essential to guide therapeutic decisions. Accumulating further case-based evidence will be critical to establishing standardized diagnostic strategies and improving outcomes in patients with this rare and aggressive tumor.
